# Meetings of Societies

**Published:** 1924-04

**Authors:** 


### MEETINGS OF SOCIETIES.

### Bristol Medico-Chirurgical Society.

*February 13th, 1924.*

Mr. Ackland read a paper on "**Toxic Myocarditis due to Dental Caries,**" and showed the patient, a medical man, who took part in the discussion (*vide* pp. 79 to 82).

Mr. A. R. Short read a paper on "**Carcinoma of the Breast**" (*vide* pp. 64 to 79).

Mr. Claremont demonstrated a portable electric lamp for fixing to the patient's head to illuminate the mouth for dental work, and suggested that it might be useful in other operations in the mouth and in the ear.

*March 12th, 1924.*

**Cases were shown as suitable for treatment by Radium** by Mr. Hey Groves, Dr. Patrick Watson-Williams and Mr. Duncan Wood.

Mr. A. E. Hayward Pinch, F.R.C.S., gave an address on "**Radium Therapy,**" with lantern illustrations.

Mr. Hayward Pinch's address and brief notes of the cases shown, as well as the discussion following, will appear in the July issue of the *Journal*.

